# Diseases of the Liver

**Published:** 1888-10

**Authors:** 


**Diseases of the Liver.** By PROF. DUJARDIN-BEAUMETZ. Translated from the fifth French edition by E P. HURD, M. D. Detroit: George S. Davis. 1888.

